# Development and validation of a prognostic nomogram based on the log odds of positive lymph nodes (LODDS) for breast cancer

**DOI:** 10.18632/oncotarget.8091

**Published:** 2016-03-15

**Authors:** Jiahuai Wen, Feng Ye, Xiaofang He, Shuaijie Li, Xiaojia Huang, Xiangsheng Xiao, Xiaoming Xie

**Affiliations:** ^1^ Department of Breast Oncology, Sun Yat-Sen University Cancer Center, State Key Laboratory of Oncology in South China, Collaborative Innovation Center for Cancer Medicine, Guangzhou, Guangdong, China

**Keywords:** breast cancer, LODDS, prognosis, nomogram, surgery

## Abstract

**Background:**

To evaluate the prognostic effect of log odds of positive lymph nodes (LODDS) and develop a nomogram for survival prediction in breast cancer patients at the time of surgery.

**Results:**

LODDS was an independent risk factor for cancer-related death in breast cancer (hazard ratio: 1.582, 95%CI: 1.190-2.104). Menopausal status, tumor size, pathological lymph node staging, estrogen receptor status and human epidermal growth factor receptor-2 status were also included in the nomogram. The calibration plots indicated optimal agreement between the nomogram prediction and actual observation. Discrimination of nomogram was superior to the seventh edition TNM staging system [C-index: 0.745 *vs.* 0.721 (*p* = 0.03) in training cohort; 0.796 *vs*. 0.726 (*p* < 0.01) in validation cohort].

**Methods:**

We retrospectively evaluated 2023 breast cancer patients from Jan 2002 to Dec 2008 at our center. The cohort was randomly divided into training cohort and validation cohort. Univariate and multivariate analyses were performed to identify prognostic factors, and nomogram was established using Cox regression model in training cohort. External validation of the nomogram was performed in the validation cohort.

**Conclusions:**

The LODDS is an independent prognostic indicator in breast cancer and the novel nomogram can provide individual prediction of cancer-specific survival and help prognostic assessment for breast cancer patients.

## INTRODUCTION

Breast cancer is the most common cancer and the leading cause of cancer death in women worldwide. According to the National Cancer Institute, more than 230,000 new cases and 40,000 deaths were reported in 2014 in the United States [1]. Although the advances in treatments of breast cancer have improved the therapeutic effect [2], local recurrences and distant metastases remain the main challenges to clinicians [3].

In breast cancer, axillary lymph nodes (LNs) assessment is crucial for the evaluation of disease severity, treatment options and prognosis assessment [4]. Increase in the number of positive LNs is independently related to increased risk of recurrence and decrease of overall survival [5, 6]. Traditionally, the National Comprehensive Cancer Network (NCCN) Guidelines recommend the number of positive LNs for axillary assessment and classify patients into different stages according to the American Joint Committee on Cancer (AJCC) tumor-node-metastasis (TNM) staging system. However, the TNM staging system does not consider the total number of LNs retrieved, and limited total LNs may cause stage migration and influence the accuracy of prognosis assessment [7, 8].

Ratio-based nodal systems were proposed as alternative tools of LN assessment in recent studies. Log odds of positive lymph nodes (LODDS), defined as the log of odds between number of positive nodes and number of negative nodes, showed superiority over the AJCC pN staging in several cancers, like gastric cancer [9], pancreatic cancer [10] and colorectal cancer [11, 12], especially when insufficient lymph nodes were retrieved. In breast cancer, the LODDS was considered as an independent prognostic factor and more reliable than the pN staging [13, 14].

Nomogram is graphical calculating tool used to quantify risk through intuitive graphs. It could provide individualized prognostic information based on the prognostic factors and be more accurate than the conventional staging systems for predicting prognosis in some cancers [15, 16].

In this study, we aimed to assess the prognostic significance of the LODDS and compare its prognostic value with pN staging in breast cancer. Additionally, a predictive postoperative nomogram based on the LODDS was developed and externally validated in breast cancer patients underwent modified radical mastectomy.

## RESULTS

### Baseline characteristics

A total of 2023 female patients with primary non-metastatic invasive breast cancer were enrolled. Then, 1494 patients were randomly selected as training cohort and 519 patients were included in the validation cohort. Patient characteristics were listed in Table 1. The mean age of the overall cohort was 49.4 years old (range 22-86 years), and 171 (8.5%) patients were under the age of 35. There were 1077 (53.2%) patients suffered regional lymph node metastases, and 305 (15.1%) patients had less than 10 total lymph nodes retrieved. Stage I, II and III accounted for 26.6%, 52.7% and 20.7% of the study cohort, respectively. Luminal subtype comprised 76.6% of total participants, and 218 (10.8%) and 254 (12.6%) were HER2 over-expressing subtype and triple-negative subtype respectively. No significant difference was observed between the training cohort and validation cohort regarding the clinicopathological factors analyzed.

**Table 1:** Clinicopathological characteristics of patients in the training and validation cohort

Characteristic	All patients (*n* =2023)	Training cohort (*n* = 1504)	Validation cohort (*n* = 519)	*p* value
Age				0.836
<35	171 (8.5)	126 (8.4)	45 (8.7)	
>35	1852 (91.5)	1378 (91.6)	474 (91.3)	
Menopause				0.457
Yes	540 (26.7)	395 (26.3)	145 (27.9)	
No	1483 (73.3)	1109 (73.7)	374 (72.1)	
Tumor type				0.630
IDC	1963 (97.0)	1461 (97.1)	502 (96.7)	
ILC	60 (3.0)	43 (2.9)	17 (3.3)	
Histologic grade				0.865
G1	76 (3.7)	56 (3.6)	20 (3.8)	
G2	1214 (60.0)	900 (60.0)	314 (60.5)	
G3	733 (36.3)	548 (36.4)	185 (35.7)	
Tumor size				0.947
T1	852 (42.1)	633 (42.1)	219 (42.2)	
T2	1068 (52.8)	793 (52.7)	275 (53.0)	
T3	103 (5.1)	78 (5.2)	25 (4.8)	
AJCC LN status				0.216
N0	946 (46.8)	712 (47.3)	234 (45.1)	
N1	553 (27.3)	411 (27.3)	142 (27.4)	
N2	308 (15.2)	215 (14.3)	93 (17.9)	
N3	216 (11.2)	166 (11.0)	50 (9.6)	
Total LN				0.455
<10	305 (15.1)	232 (15.4)	73 (14.1)	
≥10	1718 (84.9)	1272 (84.6)	446 (85.9)	
ER				0.280
Positive	1264 (62.5)	950 (63.2)	314 (60.5)	
Negative	759 (37.5)	554 (36.8)	205 (39.5)	
PR				0.364
Positive	1381 (68.3)	1035 (68.8)	346 (66.7)	
Negative	642 (31.7)	469 (31.2)	173 (33.3)	
HER-2				0.069
Positive	550 (27.2)	393 (26.1)	157 (30.3)	
Negative	1473 (72.8)	1111 (73.9)	362 (69.7)	
Cancer-specific survival				0.566
Alive	1687 (83.4)	1250 (83.1)	437 (84.2)	
Death	336 (16.6)	254 (16.9)	82 (15.8)	
LODDS (mean± SD)	−0.878 ± 0.715	−0.879 ± 0.721	−0.875 ± 0.696	0.909

Abbreviations: SD standard deviation, IDC Invasive ductal carcinoma, ILC Invasive lobular carcinoma, LN lymph node, ER Estrogen receptor, PR Progesterone receptor, HER2 Human epidermal growth factor receptor-2, LODDS log odds of positive lymph nodes

### Analysis of the prognostic impact of LODDS

The mean LODDS was −0.878 (range: −1.88 to 1.79) in the training cohort. Cancer-related death occurred in 336 (16.6%) patients during a median follow-up time of 75.1 (range 5-144) months after surgery. Table S1 listed the median survival time of different levels according to the value of LODDS with an interval of 0.5. Patients with similar prognosis were classified into four groups: LODDS1 (LODDS≤ −1.00), LODDS2 (−1.00 < LODDS ≤ 0), LODDS3 (0 < LODDS ≤ 1.5) and LODDS4 (LODDS > 1.5). The 10-year survival rates of patients in LODDS1, LODDS2, LODDS3 and LODDS4 were 87.1%, 74.0%, 48.2% and 14.4% respectively (*p* < 0.001).

In univariate analysis, age, menopausal status, histologic grade, tumor size, hormone receptor statuses, HER2 status, pN stages and LODDS were associated with cancer-specific survival of breast cancer patients (Table 2, all *p* < 0.05). In the step 1 and step 2 multivariate survival analyses, pN stages and LODDS were identified as independent prognostic factors respectively. In the step 3 multivariate survival analysis, pN stages and LODDS remained statistically significant in the same model (Table 2, all *p* < 0.05). Other independent prognostic factors included menopausal status, tumor size, ER status and HER2 status.

**Table 2:** Univariate and multivariate analyses on CSS in breast cancer patients

Variable	Univariate analysis		Step 1 Multivariate analysis		Step 2 Multivariate analysis		Step 3 Multivariate analysis	
	HR (95%CI)	*p* value	HR (95%CI)	*p* value	HR (95%CI)	*p* value	HR (95%CI)	*p* value
Age	0.649 (0.465-0.906)	0.011	0.746 (0.528-1.055)	0.098	0.737 (0.521-1.042)	0.084	0.751 (0.531-1.061)	0.105
Menopausal status	0.671 (0.527-0.855)	0.003	0.675 (0.530-0.861)	0.002	0.690 (0.541-0.880)	0.003	0.687 (0.539-0.876)	0.002
Tumor type	0.657 (0.311-1.388)	0.238	0.583 (0.272-1.250)	0.166	0.645 (0.302-1.380)	0.259	0.604 (0.282-1.293)	0.194
Histologic grade	1.244 (1.102-1.406)	<0.001	1.116 (0.977-1.274)	0.106	1.090 (0.956-1.244)	0.197	1.099 (0.962-1.254)	0.165
Tumor size	1.823 (1.519-2.188)	<0.001	1.323 (1.098-1.594)	0.003	1.343 (1.114-1.619)	0.002	1.311 (1.088-1.580)	0.004
ER	0.572 (0.462-0.709)	<0.001	0.680 (0.523-0.885)	0.004	0.649 (0.499-0.844)	0.001	0.661 (0.508-0.860)	0.002
PR	0.642 (0.517-0.799)	<0.001	0.938 (0.727-1.210)	0.623	0.923 (0.716-1.190)	0.537	0.928 (0.720-1.196)	0.564
HER2	2.021 (1.626-2.511)	<0.001	2.021 (1.626-2.511)	0.003	1.483 (1.164-1.890)	0.001	1.452 (1.139-1.853)	0.003
pN status	1.999 (1.816-2.201)	<0.001	1.439 (1.128-1.836)	0.003	-	-	1.428 (1.162-1.755)	0.001
LODDS	2.528 (2.223-2.874)	<0.001	-	-	2.429 (2.124-2.778)	<0.001	1.582 (1.190-2.104)	0.002

Abbreviations: CSS cancer-specific survival, HR hazard ratio, CI confidence interval, ER Estrogen receptor, PR Progesterone receptor, HER2 Human epidermal growth factor receptor-2, pN pathological lymph node, LODDS log odds of positive lymph nodes

Subgroup analysis indicated that pN stages and LODDS were both independently associated with the prognosis of breast cancer in both the retrieved LNs<10 and ≥10 subgroups (Table 3, all *p* < 0.05). The corresponding AUCs for pN and LODDS were 0.715 (95% CI: 0.683-0.746) and 0.709 (95% CI: 0.694-0.734) respectively, indicating that pN stages may have better discrimination capability than the LODDS, although the difference was not significant (*p* = 0.408). PN stages manifested superior model fitness over the LODDS with smaller −2LLR and AIC. In the subgroup of more than 10 lymph nodes-retrieved, the pN stages remained superior model fitness. However, the LODDS fitted the model better than the pN stages when limited lymph nodes were obtained (Table 3).

**Table 3:** Subgroup multivariate analysis and prognostic performance of pN staging and LODDS

Lymph nodes retrieved	Category	HR (95%CI)^a^	*p* value	−2LLR	AIC
All patients	pN	1.439 (1.128-1.836)	<0.001	2352.45	4706.90
	LODDS	2.429 (2.124-2.778)	<0.001	2356.10	4715.23
Lymph nodes ≥10	pN	1.920 (1.049-1.558)	<0.001	2048.12	4099.65
	LODDS	2.489 (2.159-2.869)	<0.001	2051.37	4104.91
Lymph nodes <10	pN	1.777 (1.156-2.734)	<0.001	189.94	381.88
	LODDS	2.018 (1.293-3.150)	<0.001	188.78	379.56

a Either lymph-nodes staging method was incorporated in the multivariate analysis respectively

Abbreviations: HR hazard ratio, CI confidence interval, LLR log-likelihood ratio, AIC Akaike's information criterion, pN pathological lymph node, LODDS log odds of positive lymph nodes.

### Prognostic nomogram for CSS

**Figure 1:**
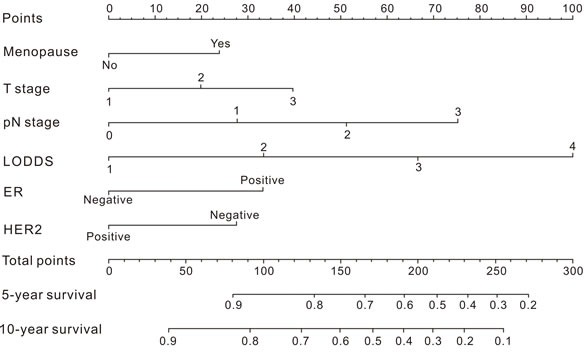
Nomogram for predicting the probability of cancer-specific survival in breast cancer patients

A nomogram based on the results of step 3 multivariate survival analysis was established (Figure 1). By summing the scores and locating on the total score scale, the estimated probability of cancer-specific survival at 5-year and 10-year could be determined.

**Figure 2:**
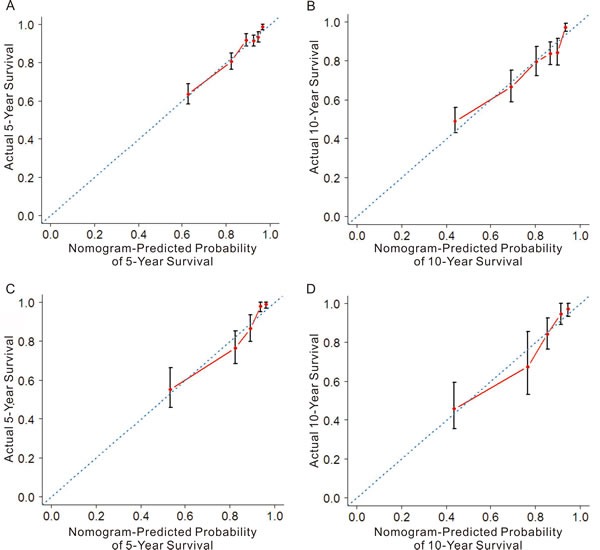
The calibration plots for predicting patient cancer-specific survival at each time point in the training cohort (A-B) and validation cohort (C-D) Nomogram-predicted CSS is plotted on the x-axis; actual CSS is plotted on the y-axis. The 45° reference line indicates the perfect predictions.

**Figure 3:**
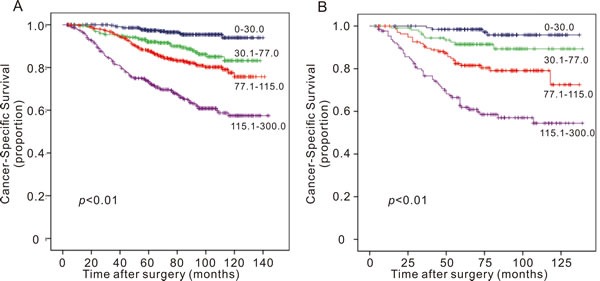
Survival curves of breast cancer patients in (A) training cohort and (B) validation cohort according to the nomogram-based classification Participants were classified into four subgroups based on the nomogram score: 0-0, 30.1-77, 77.1-115 and 115.1-300.

In the training cohort, the C-index for the established nomogram was 0.745 (95% CI: 0.721 to 0.769) and higher than that for the TNM staging system (0.721; 95% CI: 0.692 to 0.753, *p* = 0.03). In the validation cohort, the C-index for the nomogram (0.796; 95% CI: 0.756 to 0.860) was also higher than that for the TNM staging system (0.726; 95% CI: 0.665 to 0.787, *p* < 0.01). The calibration plots indicated an optimal agreement between the nomogram prediction and actual observation for 5-year and 10-year CSS in both the training cohort and validation cohort (Figure 2). After sorting by total score of nomogram, patients were classified evenly into four subgroups (total points: 0 to 30, 30.1 to 77, 77.1 to 115, 115.1 to 300). Each subgroup experienced distinct prognosis in both training cohort and validation cohort (*p* < 0.05, Figure 3). Taking the group of score 0-30 as reference, the HR for sequential subgroups were 3.105, 4.611 and 10.686 in training cohort and 3.184, 7.201 and 17.212 in the validation cohort respectively.

## DISCUSSION

In the current study, we evaluated 2023 breast cancer patients who received modified radical mastectomy and the results shown that: (1) the LODDS was an independent prognostic parameter along with the AJCC pN staging system; (2) LODDS manifested better model fitness when inadequate lymph nodes (<10) were obtained in the surgery; (3) a nomogram was established and performed well in predicting survival in breast cancer patients.

Axillary lymph node status is considered as the most important prognostic factor in breast cancer patients. In the AJCC TNM staging system, the pN stages are defined as N0 (no positive lymph node), N1 (1-3 lymph nodes), N2 (4-9 lymph nodes) and N3 (≥10 lymph nodes), but the accuracy of pN staging may be lowered by the limited number of lymph nodes retrieved, called stage migration [17-19].

Previous studies had indicated that ratio-based LN staging systems are more accurate in predicting the prognosis when limited LNs are retrieved [20]. LODDS was reported to provide more accurate information of lymph node status and be superior to TNM staging systems. In the present study, survival rates between the LODDS 1 to 4 were significantly different (*p* < 0.05). When considering the total number of retrieved LNs, LODDS remained independently associated with the prognosis of breast cancer patients in the limited (<10) and sufficient (≥10) retrieved-LNs groups respectively (both *p* <0.05). AIC and −2LLR were calculated to evaluate the model fitness, and LODDS only manifested better model fitness than pN staging in the limited retrieved-LNs group.

Since the LODDS is determined by the number of positive and negative LNs, more metastatic LNs indicate higher LODDS and pN staging. However, patient with same number of positive LNs may have various LODDS levels when considering the total number of retrieved LNs. Several studies have investigated the prognostic effect of negative LNs and proposed that negative LN should be considered when predicting the clinical outcomes in cancer patients [22, 23]. Thus, the LODDS could improve the accuracy of LN assessment and better stratify patients with different prognosis when inadequate LNs were retrieved. On the other hand, LODDS may also underestimate the LN status when the ratio of positive and negative LNs is same. Thus we consider the LODDS as a complement to pN staging and could work together to further improve the accuracy of lymph node assessment in breast cancer patients.

Nomogram has been considered as an effective tool to quantify risks and maximize predictive accuracy in several cancer types [24-26]. The nomogram illustrated that the LODDS had major contribution to the prognosis of breast cancer patients, followed by pN stages. Although the pN staging system had a slightly better model fitness than the LODDS, the latter manifested higher hazard ratio, which was the possible reason for the largest impact on the nomogram. Calibration plots showed the optimal agreement of survival rates between the prediction and actual observation in the training cohort and validation cohort respectively, indicating the reliability of the established nomogram in the current study. Discrimination capability was assessed by the C-index, and the nomogram showed better performance of prognosis prediction with higher C-index when compared with the AJCC TNM staging system.

There were several limitations in our study. Firstly, this was a single-center retrospective study and multicentric prospective studies are needed to reduce selection bias and prove the clinical value of the nomogram scoring system. Secondly, although the prediction accuracy of the nomogram scoring system exceeded that of the TNM staging system, there were 21.5%-25.5% of predictions made incorrectly. Tumor-related markers could be incorporated into the nomogram for further improvement of the prediction accuracy.

In conclusion, our study demonstrated that LODDS was independently associated with the prognosis of breast cancer patients, and it performed well when limited lymph nodes were obtained. Furthermore, we established and validated a novel nomogram to predict the risk of cancer-related death with better discrimination property than the traditional TNM staging system.

## MATERIALS AND METHODS

### Study population

Consecutive patients histologically diagnosed as breast cancer between January 2002 and December 2008 in Sun Yat-sen University Cancer Center (SYSUCC) were retrospectively reviewed. Inclusion criteria were as follows: (1) received modified radical mastectomy; (2) female; (3) pathological diagnosed as invasive ductal carcinoma (IDC) or invasive lobular carcinoma (ILC). Exclusion criteria included: (1) received neoadjuvant therapy before surgery; (2) had surgical treatment before admission; (3) with previous or coexisting cancers other than breast cancer; (4) confirmed metastasis; (5) tumor invaded to the chest wall or to the skin; (6) not enough data could be extracted. All patients were followed up to March 31 2015 or date of deaths. Every enrolled patient was randomly allocated as “training” or “validation” at the ratio of 3:1 and 75% of participants were selected as the training cohort. The remaining 25% of patients were grouped as the validation cohort.

### Clinical data collection

Clinical characteristics collected for subsequent analysis included age, menstrual status, pathological diagnosis, histologic grade, tumor size, number of positive lymph nodes, number of total retrieved lymph nodes, hormone receptor and human epidermal growth factor receptor-2 (HER2) status and date of last follow-up or death. The clinical stages were classified according to the AJCC TNM staging system (7^th^ edition). The intrinsic subtypes were as follow: Luminal A (estrogen receptor (ER) +, progesterone receptor (PR) +, HER2 - and Ki-67≤14%), Luminal B (ER+ and HER2+ or Ki-67> 14%), HER2 over-expressing (ER-, PR-, HER2+) and triple-negative breast cancer (ER-, PR-, HER2-). HER2 positive was defined as “3+” in immunohistochemical test or “positive” in HER2 fluorescence in situ hybridization test. The follow-up of patients was started after the breast cancer surgery and performed through out-patient medical records, telephone or letters by *Department of Follow-up & Medical Record Management*.

### Statistical analyses

Categorical data were described using numbers and percentages, and Chi square tests were performed to examine the differences between groups. LODDS was defined as $\log\frac{\text{pLn}+0.5}{\text{tLN}-\text{pLN}+0.5}$, where pLN is the number of positive lymph nodes and tLN is the total number of retrieved lymph nodes. 0.5 was added to both the numerator and denominator to avoid infinite singularity [27]. The primary endpoint assessed was cancer-specific survival (CSS), calculated from the time of pathological diagnosis to the date of cancer-related death or last follow-up. Intervals of LODDS in classification were determined by comparing median survival time according to LODDS with an interval of 0.5 and combining patients with similar prognosis. Univariate analysis and 3-steps multivariate analyses (Cox proportional hazards model) were performed to identify the independent variables associated with CSS. In step 1 and step 2 multivariate analyses, pN stages and LODDS status were included respectively, and both pN stages and LODDS status were included in step 3 multivariate analysis. Survival analyses were performed in the subgroups based on the number of retrieved lymph nodes (<10 or ≥ 10). Hazard ratios (HRs) and 95% confidence intervals (CIs) were calculated from the Cox regression model. Akaike's information criterion (AIC) and −2 log-likelihood ratio (−2LLR) within the Cox regression model were calculated to compare the model fitness between different lymph node staging systems.

A nomogram was developed based on the results of multivariate analysis. Backward step-down process was performed with AIC as a stopping rule [28]. 1,000 bootstrap resamples were used for internal validation of the training cohort and the external validation was performed by applying the nomogram to the validation cohort. Concordance index (C-index) was calculated for the evaluation of the performance of nomogram and the comparison with AJCC staging system. Calibration of the nomogram was performed by comparing the predicted survival with the observed survival in both the training cohort and validation cohort. SPSS (version 19.0, Chicago, IL, USA) and R software (version 3.0.1) with the survival and rms package were used for statistical analysis. A two-tailed *p* value <0.05 was considered statistically significant.

## SUPPLEMENTARY MATERIAL TABLE


