# Supplementary material for: Development and validation of a prognostic nomogram based on the log odds of positive lymph nodes (LODDS) for breast cancer
Source: Oncotarget. 2016 Mar 15;7(15):21046–53. doi: 10.18632/oncotarget.8091 (PMC4991511; doi:10.18632/oncotarget.8091)
Supplement: Supplementary file 1 [file oncotarget-07-21046-s001.pdf]

Development and validation of a prognostic nomogram based on the log odds of positive lymph nodes (LODDS) for breast cancer

Supplementary Material

Table S1: Cancer-specific survival rate of breast cancer patients according to the value of LODDS with the interval of 0.5 (ranging from -1.88 to 1.79).

LODDS	Median survival time (months) (95%CI)	5-year CSS rate (%)	10-year CSS rate (%)	*p*[a] value
LODDS≤ -1.50	129.8 (126.9- 132.8)	93.7	90.7	0.404
-1.50 < LODDS≤ -1.00	129.6 (127.2- 132.0)	93.3	85.7	<0.001
-1.00 < LODDS≤ -0.50	123.9 (119.8- 128.1)	88.0	75.6	0.066
-0.50 < LODDS< 0	118.9 (113.2- 124.7)	81.8	72.6	<0.001
0 < LODDS ≤ 0.50	92.7 (84.1- 101.3)	62.9	50.3	0.415
0.50 < LODDS ≤ 1.00	83.5 (71.2- 95.8)	59.5	47.0	0.730
1.00 < LODDS ≤ 1.50	76.1 (63.5- 88.6)	65.9	41.9	0.038
LODDS > 1.5	56.4 (35.9-76.9)	28.7	14.4	-

[a] Compared between adjacent groups and *p* value <0.05 was considered significant.

Abbreviation: LODDS *log odds of positive lymph nodes*, CSS *cancer-specific survival*, CI *confidence interval*
